# Spatial distribution of fine-grained floodplain deposits and anthropogenic materials based on official borehole data in the floodplain of Leipzig, Germany

**DOI:** 10.1016/j.dib.2025.111275

**Published:** 2025-01-04

**Authors:** Nele Graubner, Johannes Schmidt

**Affiliations:** a Institute for Geography, Leipzig University, Johannisallee 19a, Leipzig, 04103, Germany; b Historical Anthropospheres working group, LeipzigLab, Leipzig University, Strasse des 17. Juni 2, Leipzig, 04109, Germany

**Keywords:** GIS, Sediments, Central Europe, Stratigraphy, Modelling, Fluvial geomorphology

## Abstract

This data set includes the spatial model of the thickness and distribution of fine-grained floodplain deposits in the Leipzig floodplain area. The data set originates from borehole records provided by the Saxon State Office for Environment, Agriculture, and Geology [1]. The data processing involved the categorization of the stratigraphic descriptions of the borehole logs. For that, a methodology was implemented to categorize those into 6 broader classifications (sand, gravel, clay, anthropogenic sediments, fine-grained/organic sediments and others) with 33 sub-categories. Subsequently, the stratigraphic layers were analysed to determine the depth and thickness of the fine-grained floodplain deposits, as well as the distribution of anthropogenic material. The data set was filtered, with the condition that each borehole log has at least one clayey layer and a gravel layer of at least 0.7 m thickness and, later, interpolated to present a complete spatial model for the research area. The final data set is based on 3,414 data points (data collection covers the period: 1852 to 2018) within the Leipzig floodplain and offers significant resource for future interdisciplinary research into the natural and anthropogenic history of the Leipzig's floodplains, offering valuable information for more detailed analyses and more precise modelling of fine-grained floodplain deposit distribution in the Leipzig floodplain area.

Specifications Table

**Table:** 

Subject	Stratigraphy
Specific subject area	Spatial data set of floodplain deposits.
Type of data	Shapefile (.shp) and tif-files (.tif); Supporting material (ReadME-file as .txt) Data was filtered and processed.
Data collection	Soil samples were collected by the Saxon State Office for Environment, Agriculture and Geology [1]. During the data analysis, the data set was filtered and categorized using a total of 3414 borehole logs to model the spatial distribution of fine-grained floodplain deposits.
Data source location	Leipzig is located in Eastern Germany. Data were collected in the Saxon borehole register of the Saxon State Office for Environment, Agriculture and Geology [1]. The soil data covers the area of the Leipzig floodplain with a coordinate extension N 51.266076 E 12.185626 to N 51.400984 E 12.536876 (EPSG: 25,833). Exact locations (Northing and Easting) are presented in the data for every single borehole log.
Data accessibility	Repository name: PANGAEA Data identification number: 10.1594/PANGAEA.973646 Direct URL to data: https://doi.pangaea.de/10.1594/PANGAEA.973646 The data was published as a data set in PANGAEA: Graubner, N. Schmidt, J. (2024) Spatial distribution of fine-grained floodplain deposits and anthropogenic materials based on official borehole data in the floodplain of Leipzig, Germany [dataset]. The raw data was provided by the Saxon State Office for Environment, Agriculture and Geology [1]. The R-script with the entire procedure, from data filtering and processing to interpolation, is available upon request from the corresponding author.

## 1 Value of the Data

• This data set presents the first available spatial model on subsurface stratigraphies of the Leipzig floodplain. It offers valuable information on the distribution and thickness of fine-grained floodplain deposits on a regional scale. Due to the heterogeneity of the layer descriptions in the original raw data, the processed data provide an overview of the diversity of the fine-grained floodplain deposits in the Leipzig floodplain with a homogenized categorization. Therefore, the study provides a methodological approach that can act as a guideline of homogenizing and modelling of Holocene subsurface information from official databases. The spatial distribution of anthropogenic materials and its quantification [2] in the floodplain is a relevant factor of (sub-)recent geomorphological processes in the course of the Anthropocene [3].
• Understanding the deposition system within a floodplain is crucial for geomorphological assessments, as it shapes both the current landscape and potential future changes. Floodplains are dynamic environments where sediment deposition directly influences soil fertility, habitat diversity, and flood risk management.
• The model presents an opportunity for geomorphologists to conduct an in-depth examination of varying thicknesses in fine-grained floodplain deposits within the Leipzig floodplain [4]. Furthermore, it facilitates the study of historical river migration patterns [5], the reconstruction of floodplain environments, and an exploration of human-environment interactions during the Holocene epoch [6]. The data are important for future research on Holocene sediments in the area of Leipzig and for the geomorphological classification of floodplain sediments as relevant archives of human-environment relationships.
• A comprehensive high-resolution sedimentary archive from the Holocene epoch, focusing on fine-grained floodplain deposits within the Leipzig floodplain, remains absent. Nevertheless, the historical examination of human-environmental interactions is anticipated to yield significant insights into the drivers of processes and their ecological consequences [8]. The depositional thickness layer, as presented in this study, holds potential for identifying promising locations.
• The methodological approach shows the potential of using official drilling databases for geomorphological research, particularly of Holocene deposits in the (peri‑)urban context [4,2]. The generated data set will serve in the future for methodological comparison and future empirical research on fluvial deposits could focus on improving data density and refining the interpolation. Furthermore, the maps and models provide valuable tools for urban- and landscape planning and management. This information helps planners identify areas at risk of flooding and design infrastructure that can handle varying infiltration capacities, enhancing resilience [9]. Additionally, these resources support sustainable landscape design by pinpointing zones suited for ecological restoration, recreational areas, or agricultural use, balancing urban needs with natural water management.
• The floodplain forest of Leipzig is strictly protected due to its high and unique biodiversity [10]. The impact of droughts and human impact through river regulations is crucial and can have a negative impact on plant health or even suppress natural species in the research area. The connection to detailed information on the subsurface, provided by our model, can help to understand the influence of heterogeneous subsurface conditions on biodiversity.

## 2 Background

**Fig. 1:**
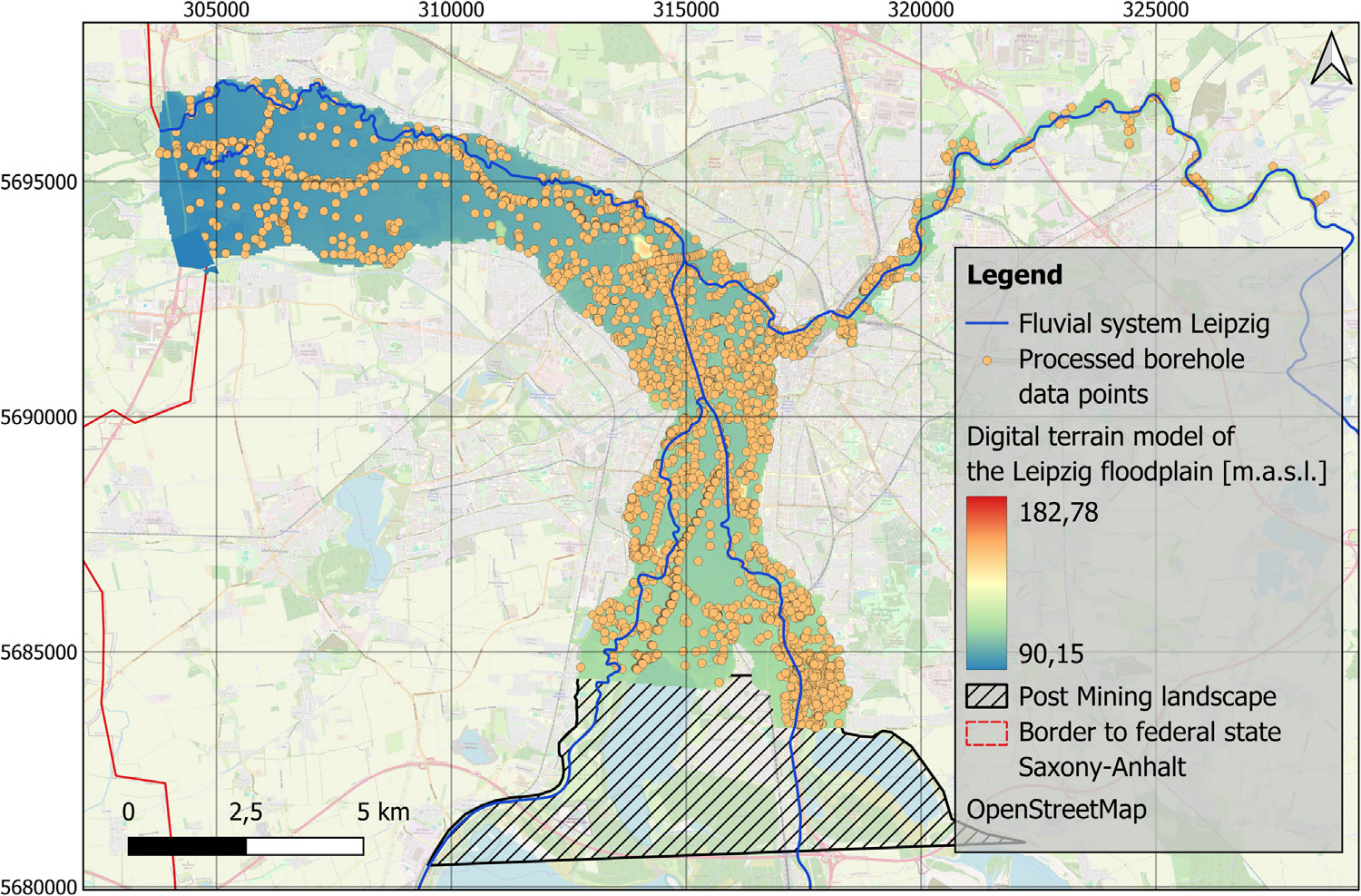
Research area of the floodplain area in Leipzig, Germany with data points of processed borehole data by the Saxon State Office for Environment, Agriculture and Geology [1]. EPSG: 25833. Background map: OSM basemap.

Understanding human-environment relationships in the past is generally important for evaluating and assessing the Anthropocene concept [11,3]. Specifically, the river landscapes of Central Europe represent areas that were heavily used and shaped by humans even in pre-industrial times. For a deeper understanding of floodplains as socio-natural sites [6], quantitative data are particularly valuable [12]. Spatial distributions of sediment units within floodplains enable a comprehensive understanding of river dynamics over time [13]. Studies of Holocene floodplain deposits have contributed to the building of a generalized model of the distribution, characteristics and thickness of fine-grained floodplain deposits [14,7]. Thanks to the official borehole database [1], a large stratigraphic data set is available for the Leipzig floodplain region. The aim was to provide an overview of the distribution of fine-grained deposits for the floodplain area in Leipzig (Fig. 1).

## 3 Data Description

**Fig. 2:**
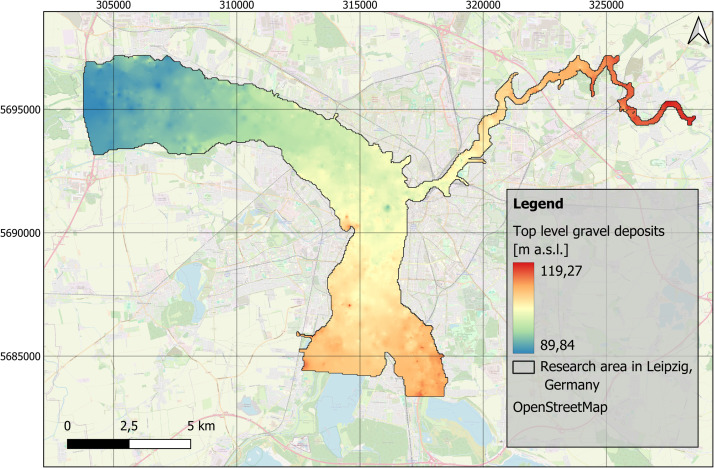
Distribution of the top level of fluvial gravels in the research area in Leipzig, Germany. EPSG: 25833. Background map: OSM basemap.

**Fig. 3:**
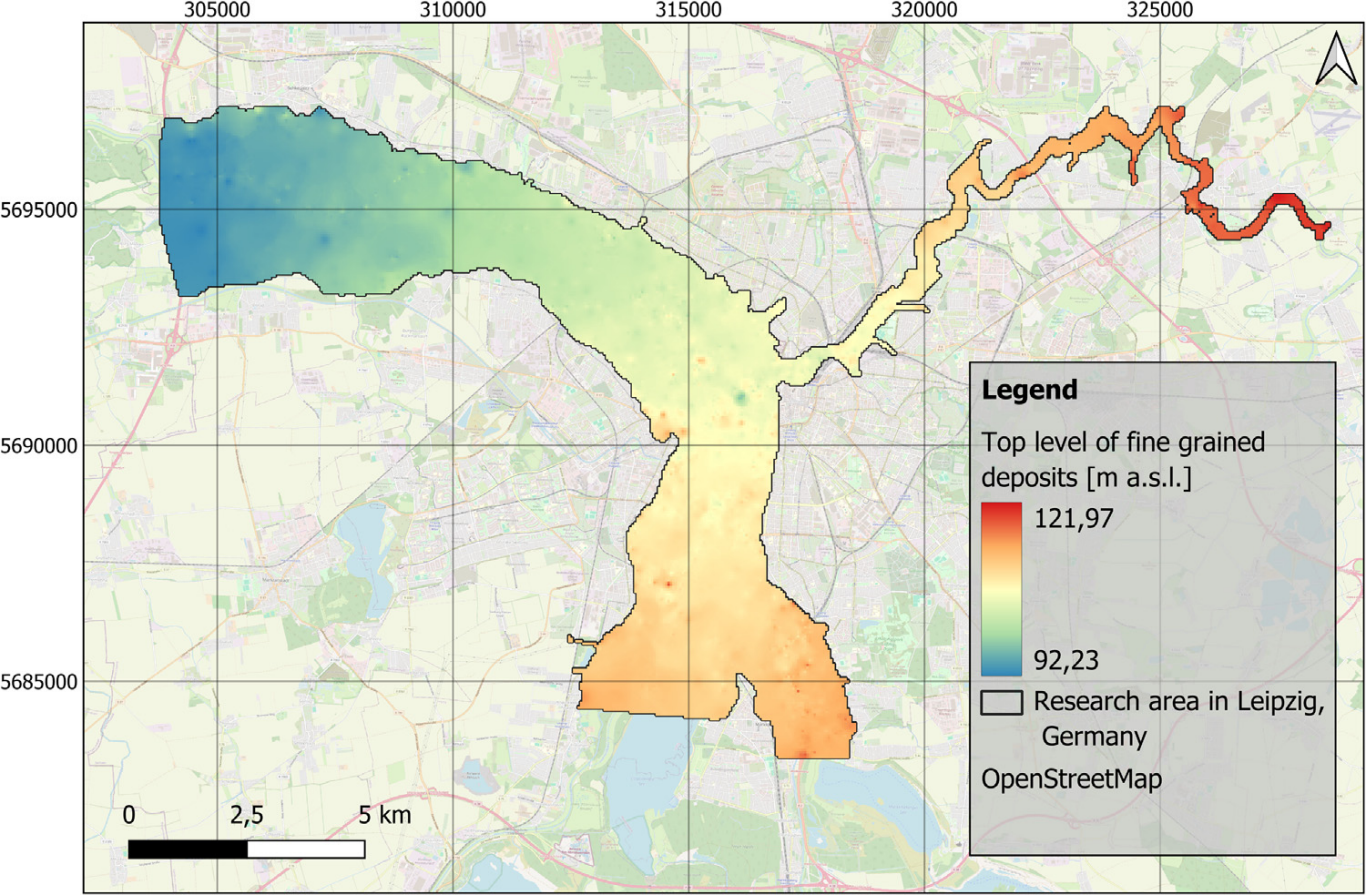
Distribution of the top level of fine-grained floodplain deposits in the research area in Leipzig, Germany. EPSG: 25833. Background map: OSM basemap.

**Fig. 4:**
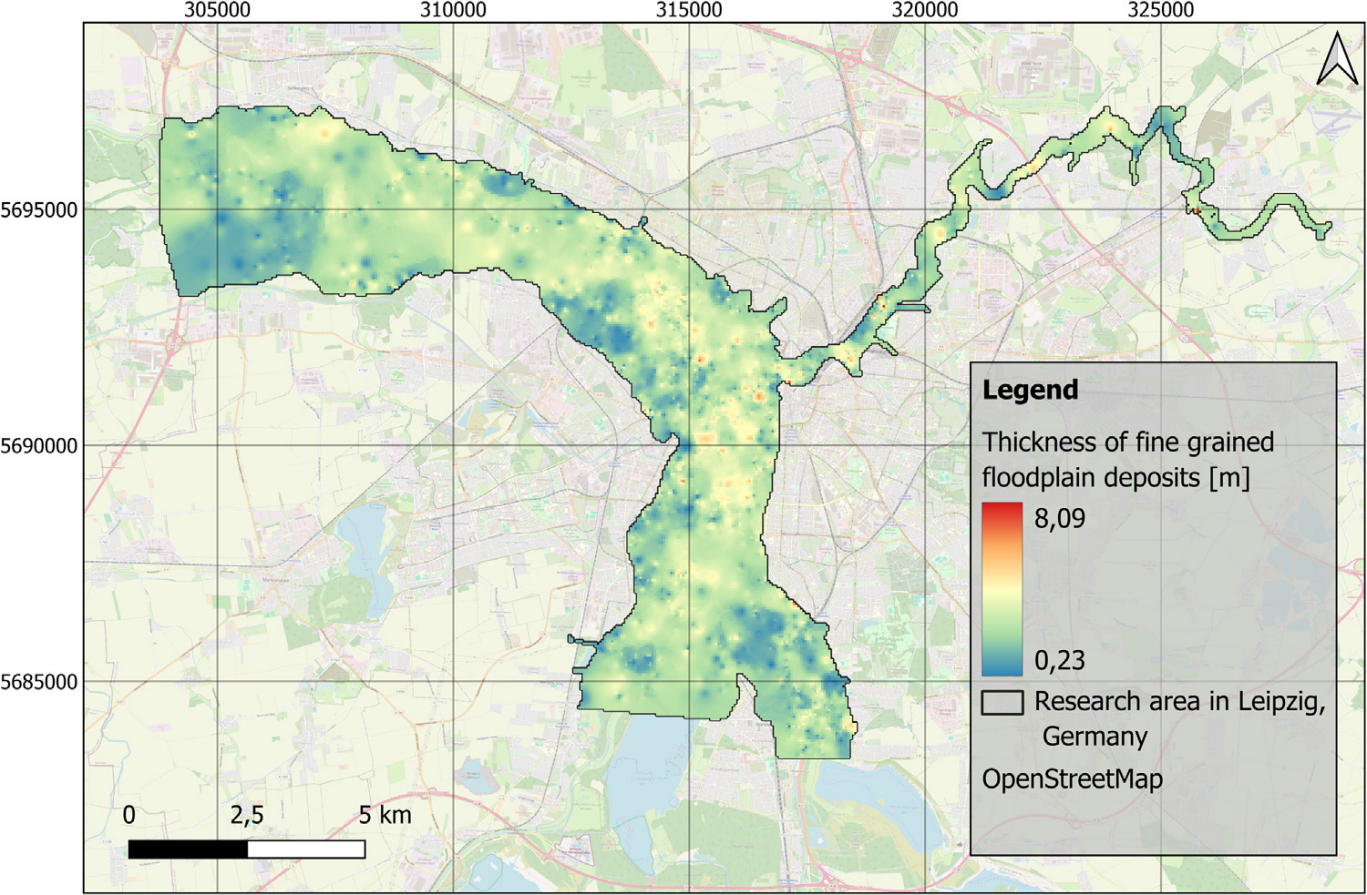
Distribution of the thickness of fine-grained floodplain deposits in the research area in Leipzig, Germany. EPSG: 25833. Background map: OSM basemap.

**Fig. 5:**
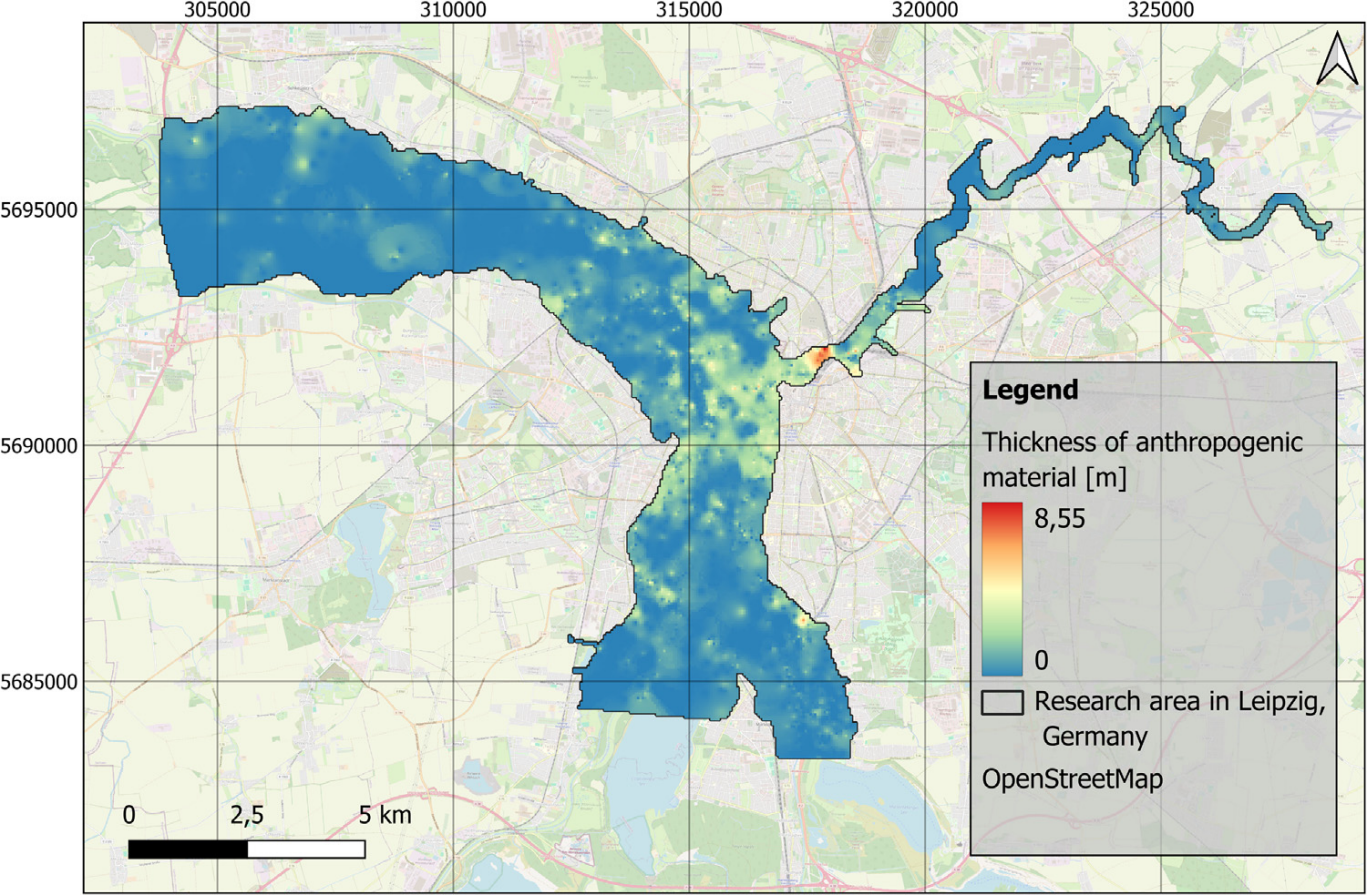
Distribution of the thickness of the anthropogenic material in the research area in Leipzig, Germany. EPSG: 25833. Background map: OSM basemap.

This data set consists of four raster layers, one shape file and one README-File (Table 1). The first raster layer refers to the interpolated level of the top layer of fluvial gravels in the research area. The raster layer is provided as a tif-file and can be presented in a map (Fig. 2). The second raster layer provides the top level of the fine-grained floodplain deposits (Fig. 3), while the third raster layer shows the distribution of the thickness of that exact layer (Fig. 4). Additionally, the stratigraphic data was used to spatially model the distribution of thickness of the anthropogenic material in the research area (Fig. 5). All raster layers have cell sizes of 50 × 50 m and are based on 3414 borehole logs (data collection covers the period: 1852 to 2018), provided by the Saxon State Office for Environment, Agriculture and Geology [1]. The processed data are provided as shape file (.shp), presenting every single borehole with easting and northing information, as well as the numerical information on the top level gravel layer, the top level fine-grained deposits and the thicknesses of the fine-grained floodplain deposits, as well as the thickness of anthropogenic material, that were used for the raster layers and maps. Furthermore, the data set includes a ReadME-file (.txt), that provides technical data description.

**Table 1:** Parameters of data set of spatial distribution of floodplain sediments in Leipzig, Germany.

1. Raster Layer “TopLevelGravel.tif”	Z-value: Top level of fluvial gravels [m a.s.l.] EPSG: 25833 - ETRS89 / UTM zone 33N Cell size: 50 × 50m
2. Raster Layer “TopLevelFineGrained.tif”	Z-value: Top level of fine-grained floodplain deposits [m a.s.l.] EPSG: 25833 - ETRS89 / UTM zone 33N Cell size: 50 × 50m
3. Raster Layer “ThicknessFineGrained.tif”	Z-value: Thickness of fine-grained floodplain deposits [m] EPSG: 25833 - ETRS89 / UTM zone 33N Cell size: 50 × 50m
4. Raster Layer “ThicknessAnthropogenicMaterial.tif”	Z-value: Thickness of anthropogenic material [m] EPSG: 25833 - ETRS89 / UTM zone 33N Cell size: 50 × 50m

## 4 Experimental Design, Materials and Methods

**Fig. 6:**
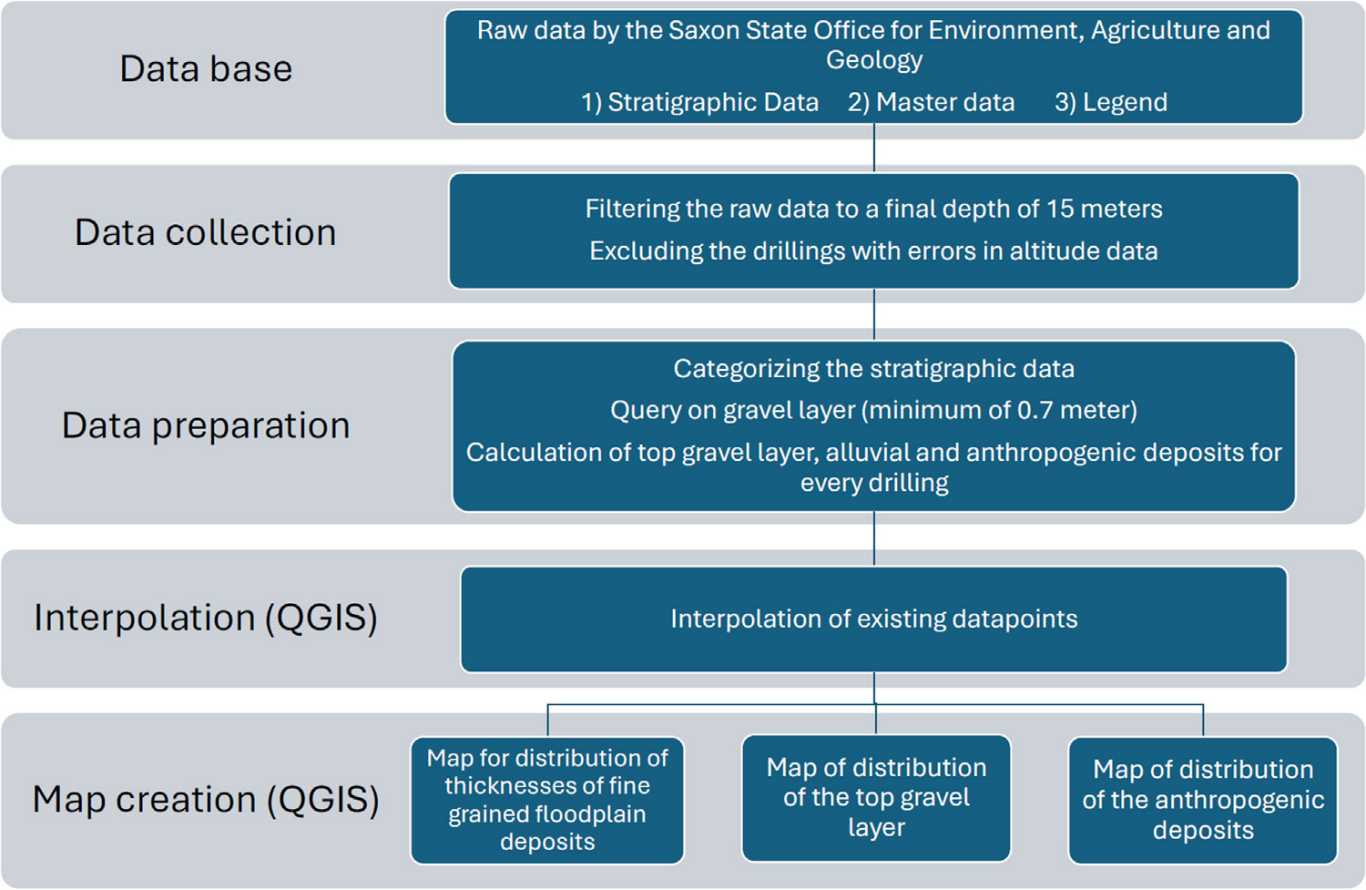
Workflow of experimental design and methods for processing the data set of spatial distribution of fine-grained floodplain deposits in Leipzig, Germany.

The workflow begins with data acquisition (Fig. 6) where raw data is gathered from the Saxon State Office for Environment, Agriculture, and Geology [1]. Next, data collection step includes filtering and cleaning of the data to ensure data consistency. The subsequent step involves the categorization of the stratigraphic data, ensuring the formation of a homogeneous and generalized borehole log data set. The interpolation step facilitates the networking of data, culminating in the generation of raster data.

### 4.1 Data acquisition

The data acquisition for this study is based on the Saxon borehole register, provided by the Saxon State Office for Environment, Agriculture, and Geology [1]. The repository consists of comprehensive borehole log data for the research area in the Leipzig floodplain, which spans an area of approximately 69 km² (Fig. 1). The data provided contain layer information up to 20 m in depth.

### 4.2 Data collection

Upon request, the Saxon State Office for Environment, Agriculture, and Geology [1] provided the borehole data organized in three tables:

• Master Data (OEGDG001): Contains 59,870 data points (total number of layers) of 10,633 boreholes, including geographic coordinates (northing and easting), borehole top level, final depth, and date of drilling.
• Layer Data (OEGSH001): Includes 45,484 layers from 10,633 borehole logs, with parameters like layer thickness, color, stratigraphic descriptions, and grain size distribution.
• Stratigraphic Legend (Pdstrat): A legend to interpret the abbreviations of stratigraphic layers, used for categorizing and generalizing the data.

In this study, this raw data set was filtered to focus on borehole logs with maximum depths of 15 m, as only these depths are relevant for the study of fine-grained floodplain deposits regarding the stratigraphy of the region [7]. The raw data by the Saxon State Office for Environment, Agriculture and Geology provided data for 10,633 boreholes. The filtering process excluded borehole logs, mostly due to incorrect or missing elevation data. Two borehole logs were excluded as they only consist of anthropogenic data, such as concrete or asphalt layers. After filtering, the data set contains 8247 boreholes.

### 4.3 Data preparation and processing

Data processing was conducted using R software (version 4.3.2) [15], where the master data and layer data were merged. The merged data set for the spatial model comprises various variables that characterize the borehole log (Table 2).

**Table 2:** Description of relevant parameters in raw data provided by the Saxon State Office for Environment, Agriculture, and Geology [1].

Abbreviation	Description
ID NR	ID number
AKBEZ	Short description of borehole
RECHTS GK	Easting
HOCH GK	Northing
UTIEF	Depth of base of each layer
MAE	Layer thickness
PET	Rock type
STRAT	Stratigraphic abbreviation
PETVERB	Stratigraphic description
HOEHE	Level of borehole log
HSYS	Elevation reference system
ENDT	Final depth of borehole
NAME	Borehole location
BZEITE	Borehole date
NNBS	Elevation of deepest point of borehole

The data processing involved the challenge to overcome the high stratigraphic description heterogeneity (45,484 layers with 12,254 unique descriptions). For that, a methodology was employed to categorize those into 6 broader classifications:

• Sand
• Gravel
• Clay
• Anthropogenic sediments
• Fine-grained or organic sediments
• Others

These broader categories were further divided into 33 subcategories (Table 3). With these categories, the borehole data were filtered on the condition of having at least one layer of sediment that would fall into the broad classification of clay, which resulted in 5937 remaining borehole logs. Additionally, all layers with anthropogenic material like concrete or asphalt were excluded.

**Table 3:** Categories with subcategories for stratigraphic descriptions of borehole data provided by the Saxon State Office for Environment, Agriculture, and Geology for further data analysis [1].

Sand	Gravel	Clay	Anthropogenic	Fine-grained or organic deposits	Others
Fine sand	Fine gravel	Alluvial sandy clay	Masonry	Mud	Rock
Medium sand	Medium Gravel	Alluvial silty clay	Road construction waste	(Calcareous, silty, organic-) Mud	Lignite
Alluvial sand	Coarse Gravel	Alluvial clay	Byproduct	Peat	Suspended clay, Loess
Sandy soil	Coarse Sand	Clay	Asphalt, Blacktop	Sludge, slag, slime	Core loss
Sandy, clayey soil	Glacial Clay, -marl, -sand	Humus	(Industrial) Ash		
		Clay (sandy, gravelly, silty)	Cement		
		Sludge (sandy, loamy, clayey, humic, sandy)	(Steel-) Concrete		
			Building material		

A top level in meters above sea level was calculated for each layer, with subtracting the thickness from the lower level of each stratigraphic layer. In cases where a borehole contained a gravel layer with a thickness of at least 0.7 m, the top of the gravel layer was recorded as a reference point, and all overlying layers were classified as fine-grained floodplain deposits, with having the anthropogenic layers excluded. The limit of 0.7 m was determined based on the existing borehole data. Many boreholes showed gravel layers, that lay in between more fine-grained layers. To determine the position of fluvial gravels under floodplain deposits, it was important to adjust the minimum gravel thickness of 0.7 m. After filtering, the data set consisted of 3414 borehole logs that met the criteria for floodplain deposits. Borehole logs that did not meet these criteria were excluded from the final data set. These excluded data either showed a gravel layer of <0.7 m or no gravel layer at all. The raw data shows a significantly higher number of layers and boreholes than the final data set (Raw data: 10,633 logs; Filtered data: 8247 logs; Processed data: 3414 logs). The reduction of borehole logs from 8247 to 3414 is due to the clipping of the data set on the spatial distribution of the floodplain in the study area and the gravel layer threshold (0.7 m).

The final result of the preparation and processing steps was a data set on borehole level, which consists of a tabular structure (Table 4) that is further processed in QGIS to create spatial models [16].

**Table 4:** Structure of the processed data set on borehole level for further analysis and modelling in GIS.

ID (ID by Saxon State Office for Environment, Agriculture and Geology)	x (East- ing)	y (Nor- thing)	z (Altitude [m a.sl.])	Top level gravel [m a.s.l.]	Top level fine- grained deposits [m a.s.l.]	Thickness fine-grained floodplain deposits [m]	Thickness anthropogenic material [m]

### 4.4 Interpolation and mapping

The stratigraphic data were imported into Quantum GIS software (version 3.36.2) [16]. To provide a full spatial model and predict sediment distribution and thicknesses in areas where borehole data were missing, the interpolation method Inverse Distance Weighting (IDW) with no smoothing was applied on this data. The interpolation was carried out by using the GRASS GIS extension (version 8.3.2), with the tool v.surf.idw. The parameters were set to 12 interpolation points, a raster cell size of 50 m, and a weighting factor of 2. During interpolation four new raster layers were created, that focus on the distribution of the top level of fluvial gravels and fine-grained floodplain deposits (Fig. 2, Fig. 3), and the distribution of the thickness of fine-grained floodplain deposits and anthropogenic material (Fig. 4, Fig. 5).

The interpolation method IDW estimates the missing data appoints in each cell by averaging values from nearby borehole logs, with the assumption that points closer together are more likely to share similar characteristics [17]. However, this method does not consider hydrological or geomorphological factors, such as the proximity to rivers, which could affect sediment distribution [18]. IDW was chosen as an interpolation method, as it uses less preconditions compared to other interpolation techniques. Therefore, our results are dependent on the chosen method and slight differences would have emerged if another method would have been used. Hence, the data set consists of the classified point data, so adapted interpolation techniques can be applied.

### 4.5 Summary of processed data

The final processed data set contains 3414 borehole logs from the period 1852 to 2018. Each log was categorized into relevant stratigraphic layers, with the positions and thicknesses of the fine-grained floodplain deposits and anthropogenic material and the top level layers are provided in the data set.

**Fig. 7:**
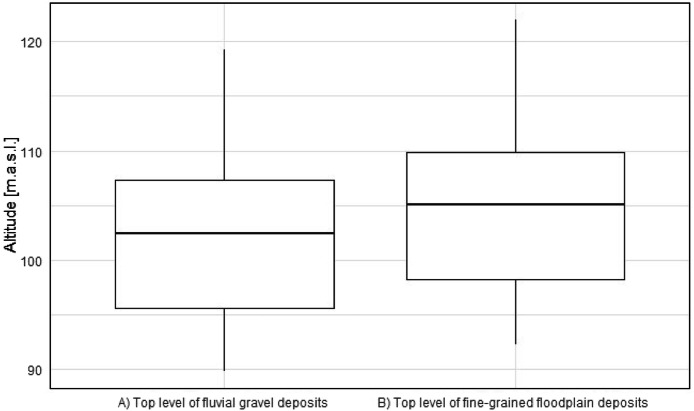
Boxplots based on the raster layers containing information on top levels for A) fluvial gravels (Fig. 2) and B) fine-grained floodplain deposits (Fig. 3).

**Fig. 8:**
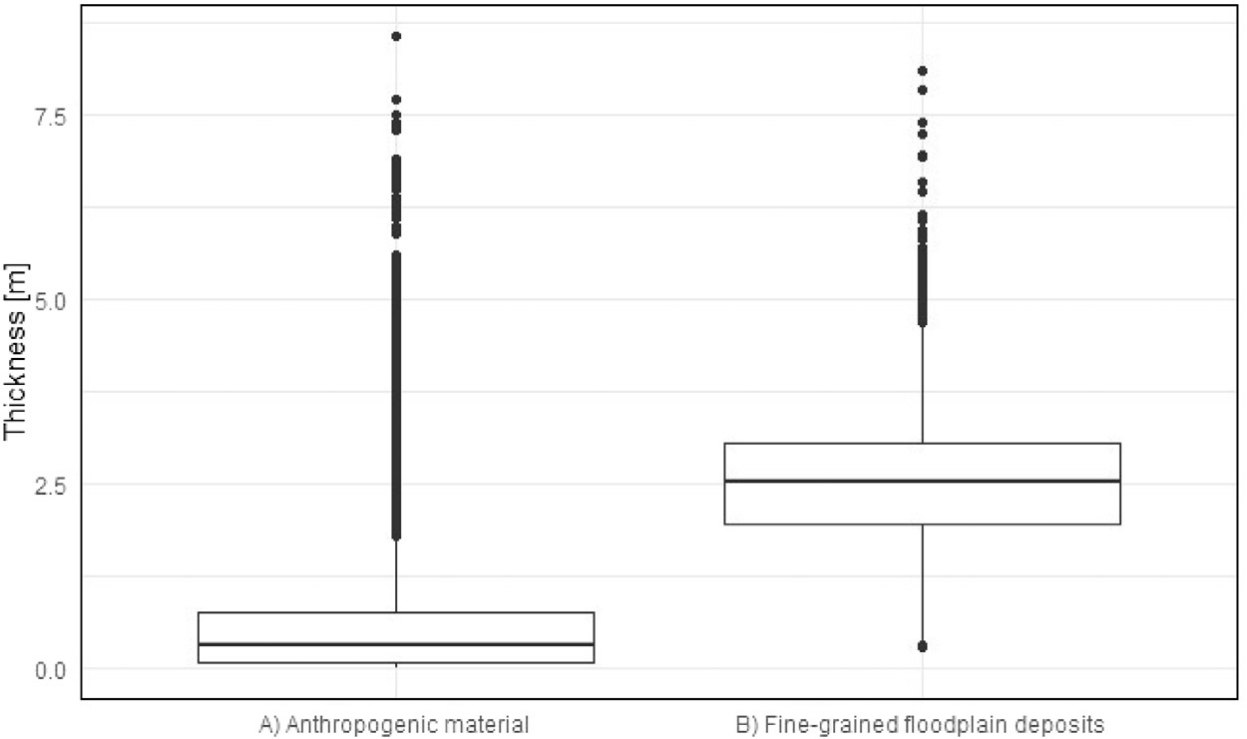
Boxplots based on the raster layers A) anthropogenic material (Fig. 5) and B) fine-grained floodplain deposits (Fig. 4).

The distribution and data range of the top level elevations (Fig. 7) and thicknesses of fine-grained deposits and anthropogenic materials (Fig. 8) e.g. show average thicknesses of fine-grained deposits of c. 2.5 m (median). This corresponds, regarding the study area (Fig. 1), to a total volume of approximately 170 Mio. m³. The anthropogenic deposits have an average thickness of 0.3 m (median), which corresponds to a total volume of 20 Mio. m³ for the study area.

## Limitations

The data set's heterogeneity, with thousands of unique stratigraphic descriptions, presented a major challenge during data processing. Various institutions and companies used different terminologies to describe sediment layers, leading to 12,254 unique descriptions from 8248 borehole logs. This inconsistency made it challenging to categorize the fine-grained floodplain deposits. Therefore, stratigraphic layers were grouped into broader classifications to generalize the data set.

The assigned categories inherently carry a degree of uncertainty that can be diminished through thorough examination of detailed stratigraphic descriptions or by comparing the data collection-based model with site-specific stratigraphic information derived from high-resolution field studies (e.g. geophysics, geoarchaeology, etc.) [4]. In the absence of such a detailed comparison, there persists a potential that broader classifications may oversimplify complex sedimentary structures [19,20].

Furthermore, it is important to note that the fine-grained alluvial deposits are free of coarse material such as gravel, but do not represent the exact distribution and thickness of only alluvial clay in the floodplains. This is due to the assignment of broader classifications and the large number of unique layer descriptions.

**Fig. 9:**
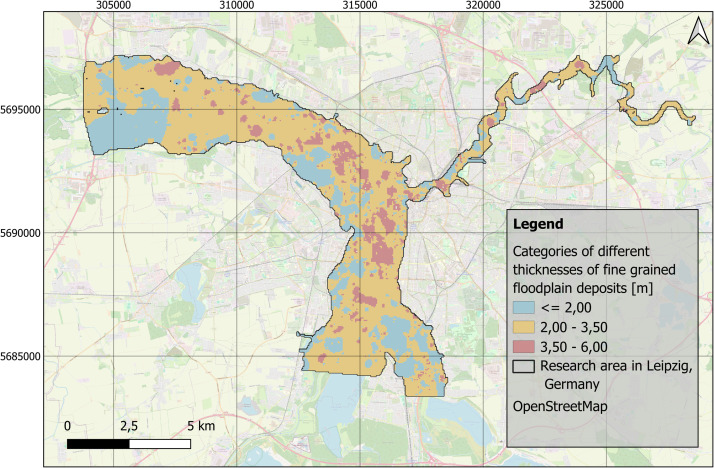
Generalized presentation of the spatial distribution of the thickness of fine-grained floodplain deposits in three distinct classes in the research area in Leipzig, Germany. EPSG: 25833. Background map: OSM basemap.

Another significant limitation relates to the interpolation method used. While Inverse Distance Weighting (IDW) estimates sediment thickness in areas with missing data, it does not account for natural small-scale boundaries or variations, like e.g. river courses. These natural features could have an influence on the sediment deposition. Further research could focus on finding an optimal way of interpolating irregularly distributed data points of sedimentary deposits [18]. As the selection of the interpolation method is challenging, one way to overcome the disadvantage of the IDW method, is to group the values of the deposits and show them in broader categories on a map in order to have an improved representation (Fig. 9).

The spatial distribution of original borehole logs varies across the study area (Fig. 1). In certain parts of the {Elster-Luppe} floodplain (northwestern part of the study area), the interpolation is based on relatively few boreholes, resulting in higher uncertainty in the sediment thickness estimates.

These challenges and limitations have implications for the quality and applicability of the data set. Therefore, the data set requires careful interpretation, particularly in areas with low data density.

## Ethics Statement

The authors have read and follow the ethical requirements for publication in Data in Brief and confirm that the current work does not involve human subjects, animal experiments, or any data collected from social media platforms.

## Credit author statement

**Nele Graubner:** Conceptualization, Methodology, Software, Formal analysis, Data curation, Investigation, Visualization, Writing - original draft, Writing - Review and editing, **Johannes Schmidt:** Conceptualization, Methodology, Funding acquisition, Writing - original draft, Writing - Review and editing, Supervision.

## Data Availability

PANGAEASpatial distribution of fine-grained floodplain deposits and anthropogenic materials based on official borehole data in the floodplain of Leipzig, Germany [dataset] (Original data).
